# Supplementary material for: Modulation of VEGF Expression and Oxidative Stress Response by Iodine Deficiency in Irradiated Cancerous and Non-Cancerous Breast Cells
Source: Int J Mol Sci. 2020 May 31;21(11):3963. doi: 10.3390/ijms21113963 (PMC7312479; doi:10.3390/ijms21113963)
Supplement: Supplementary file 1 [file ijms-21-03963-s001.pdf]

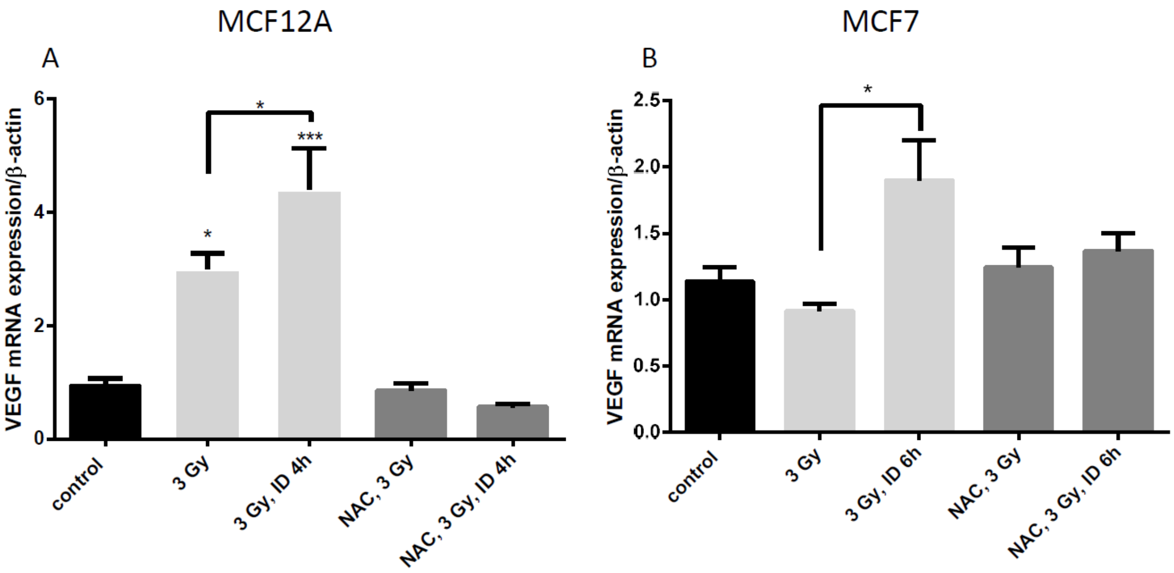

Figure S1. ID and radiations-induced VEGF mRNA up-regulation depends on ROS production in both cell lines. Cells were cultured in iodine-containing medium for 7 days. Medium was replaced by iodine-containing (control) or iodine deficient (ID) medium and cells were then irradiated with a dose of 3 Gy. Part of the cells was treated with the antioxidant NAC. Cells were harvested 4 or 6 hours (h) after medium change. VEGF mRNA expression in MCF12A (A) and MCF7 cells (B) was determined using RT-qPCR. Data are expressed as means ± SEM. P-values < 0,05 were considered as statistically significant. *P < 0.05, ***P<0.001. Statistical test: one-way ANOVA with Tukey post-hoc test. N=3, one representative experiment.
